# Supplementary material for: A comprehensive suite for extracting neuron signals across multiple sessions in one-photon calcium imaging
Source: Nat Commun. 2025 Apr 11;16:3443. doi: 10.1038/s41467-025-58817-z (PMC11992088; doi:10.1038/s41467-025-58817-z)
Supplement: Supplementary file 1 — Supplementary Information [file 41467_2025_58817_MOESM1_ESM.pdf]

Supplementary Data: CaliAli: A Comprehensive Suite for Extracting Neuron Signals Across Multiple Sessions in One-Photon Calcium Imaging.

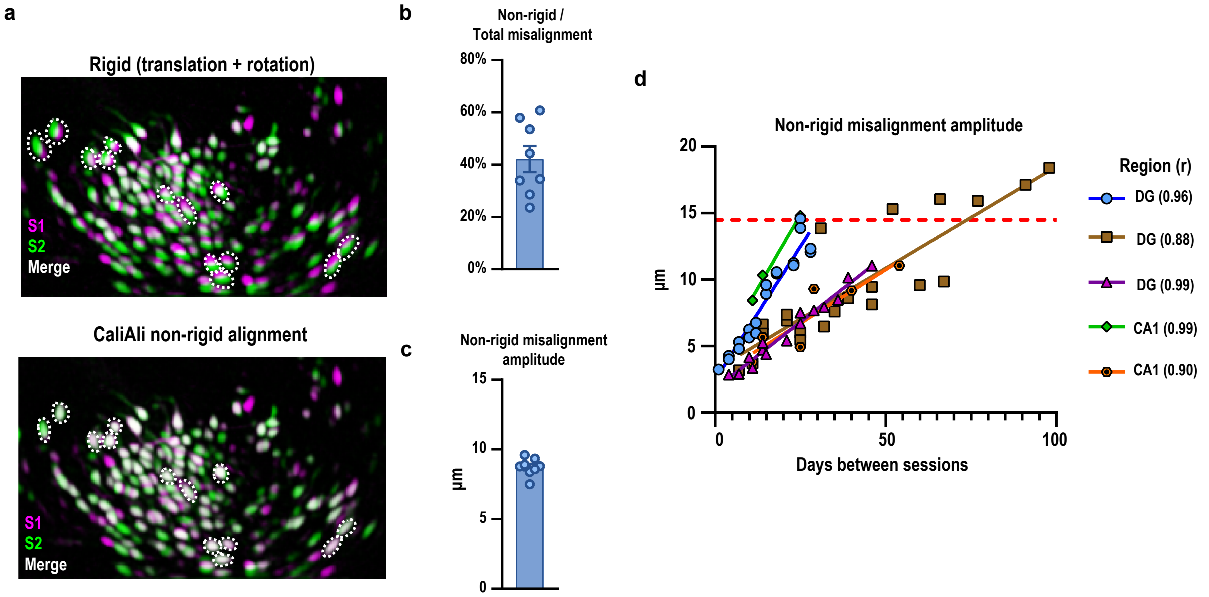

Supplementary Fig. 1 | Non-rigid misalignment increases over time. a, Overlayed correlation images of two sessions separated by a 4-day inter-session gap, aligned using translation and rotation (top) or CaliAli's non-rigid alignment (bottom). White dashed lines indicate neurons that are inaccurately aligned by translation and rotation but correctly positioned by subsequent non-rigid registration. **b,** Percentage of non-rigid misalignment in relation to the cumulative misalignment detected (i.e., translation + rotation + non-rigid). **c,** Non-rigid misalignment amplitude, defined as the 95th percentile of the distribution of non-rigid pixels displacements. Each data point corresponds to one mouse (n = 8). **d,** Misalignment amplitudes over time from dentate gyrus (DG) and CA1 recordings. Each color of datapoints represents one mouse (DG, n = 3; CA1 n = 2). Error bar = SEM.

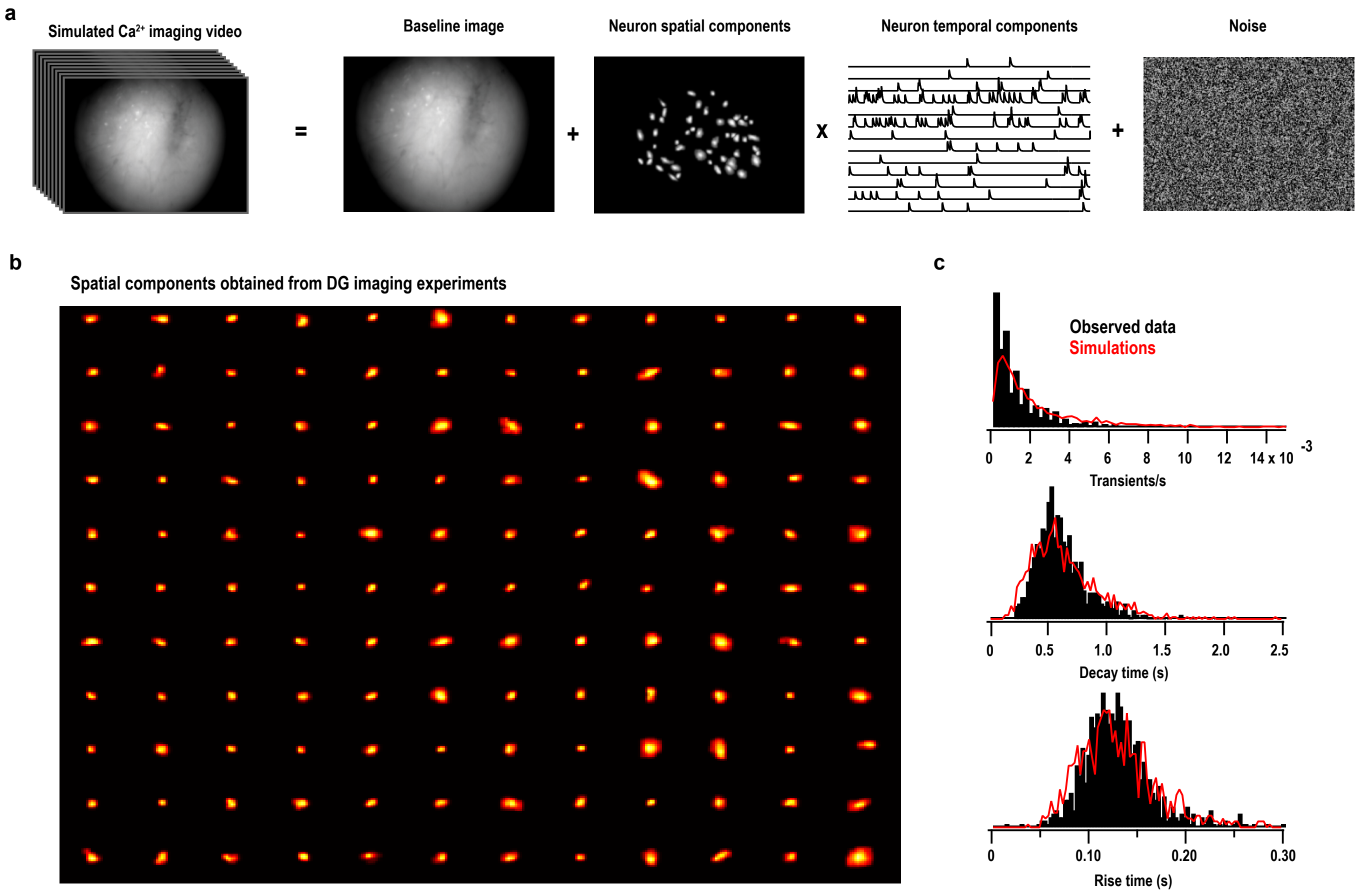

Supplementary Fig. 2 | Generating realistic simulated Ca^{2+} imaging data. **a**, Video simulations combined a constant baseline, neuron spatial and temporal component, noise, and local background fluctuations. **b**, Spatial components were simulated by randomly sampling from 1,137 DG granule cells in eight mice. **c**, Neuron mean transient rates and Ca^{2+} decay and rise times were randomly selected from a lognormal distribution with parameters estimated from actual granule neuron data.

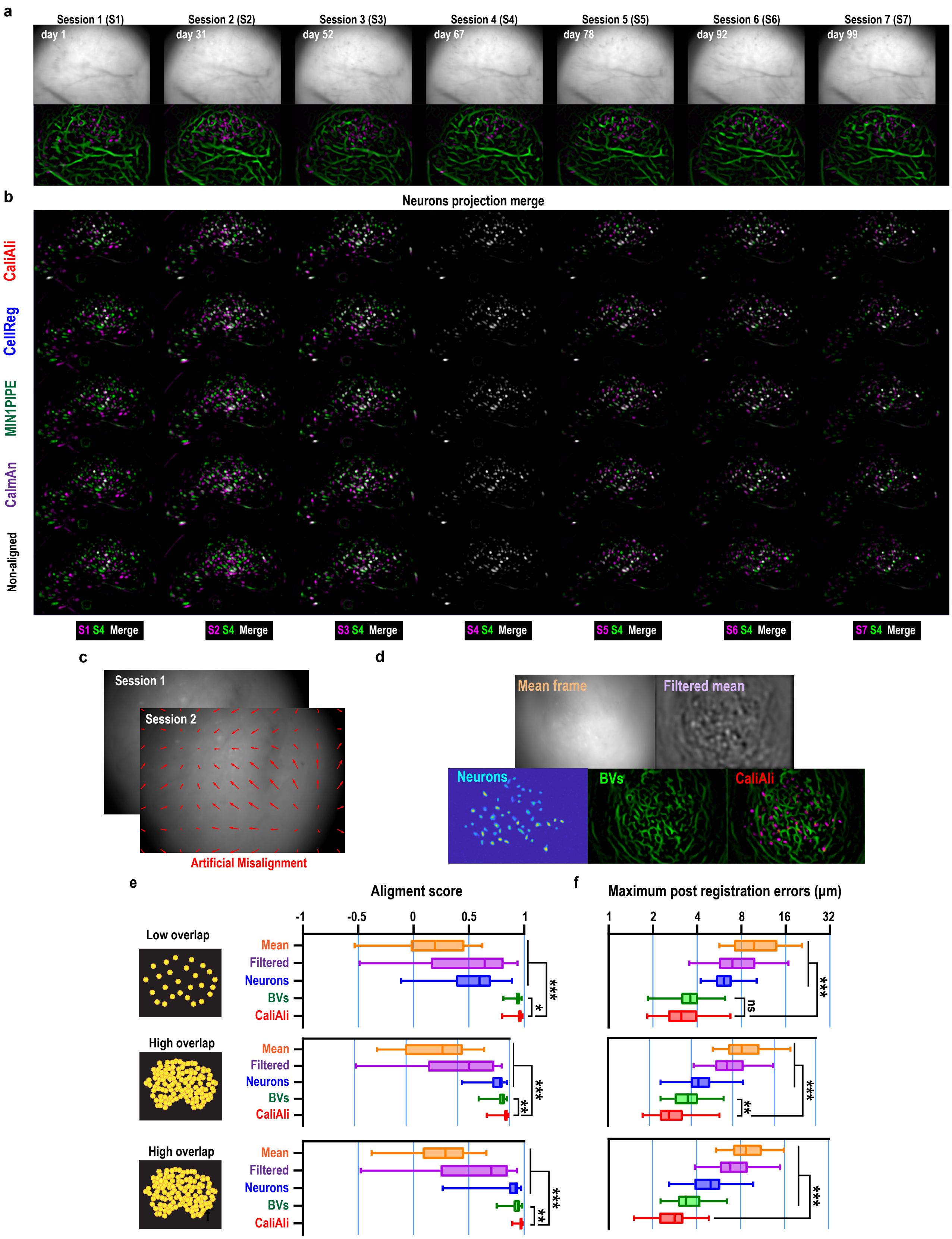

Supplementary Fig. 3 | Inter-session alignment performance using different projections. **a,** Average projections and BV +

neurons projections for seven sessions obtained across 99 days. **b**, Example of aligned neuron projections from different session pairs using different Ca^{2+} imaging packages: CellReg (Demons, neurons), MIN1PIPE (LogDemons, neurons), and CaImAn (patch phase correlation, filtered mean). Same as Fig. 2f but shown forfor all 99 days. **c,** Artificial inter-session misalignments were introduced in one of two video sessions. **d,** Different projections were evaluated to find that most suitable for inter-session misalignment correction. **e,** Alignment scores from each projection and CaliAli, which measure how well sessions are aligned by calculating the spatial correlation between the simulated deformation field (used to misalign sessions) and the inverse of the displacement field obtained from registration (used to correct these misalignments). **f,** Maximum post-registration errors with different projections. Repeated measures one-way ANOVA with Geisser-Greenhouse correction and Dunnett's multiple comparison test (each vs. CaliAli). $**p < 0.01$, $***p < 0.001$. In e and f, whiskers represent the data range.

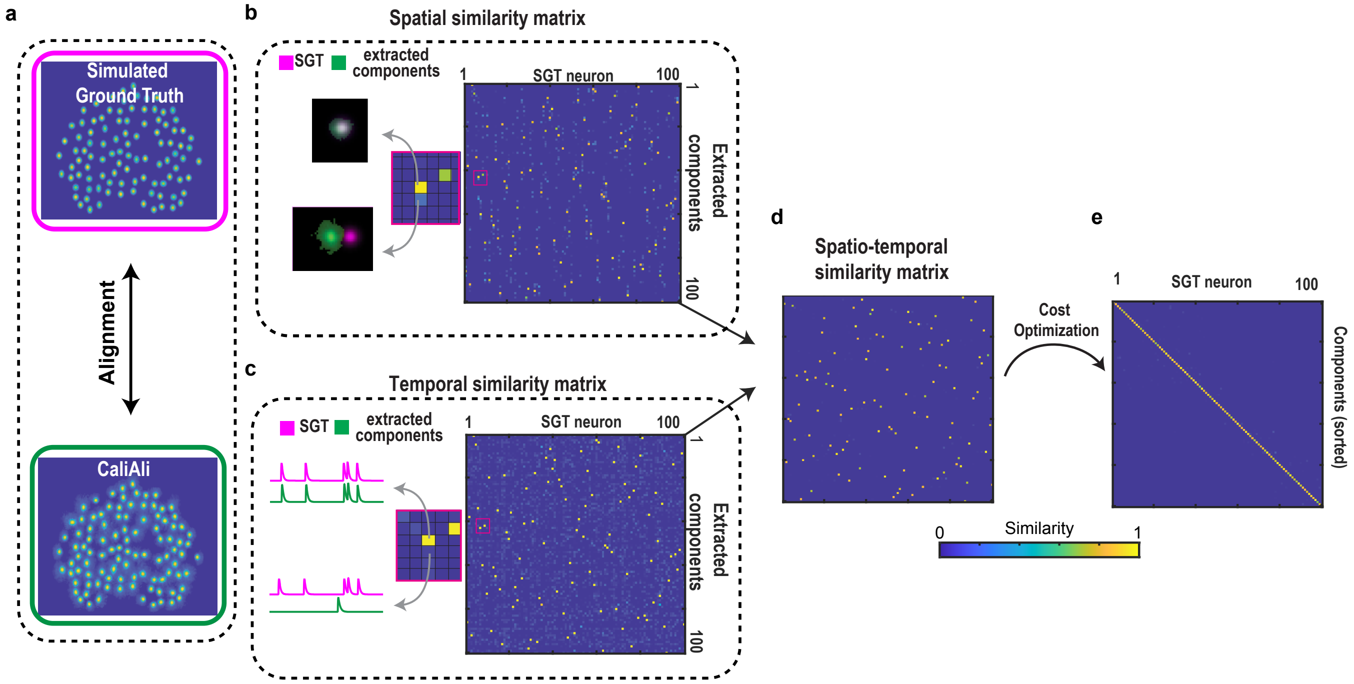

Supplementary Fig. 4 | Matching of extracted components with simulated ground truth (SGT) neurons. SGT and extracted components were matched as follows: **a**, A projection of the extracted component was aligned with the SGT footprints. **b**, A spatial similarity matrix (i.e., cosine similarity) for each combination of SGT and extracted neuron was calculated. **c**, The same was done for temporal components. **d**, An element-wise product of both matrices was calculated to create a spatiotemporal matrix. **e**, The rows and columns of the spatiotemporal matrix were permutated to find the assignment maximizing the similarity between SGT and extracted components. This linear assignment problem was solved using the matchpair function in MATLAB. This approach ensures one-to-one matching and is suitable for cases in which the number of extracted components differs from the number of SGT neurons (i.e., non-matched components have no spatiotemporal similarity). Finally, we used the temporal similarity of the matched components as a measure of tracking performance. Matched components with temporal similarity >0.8 were considered true-positives.

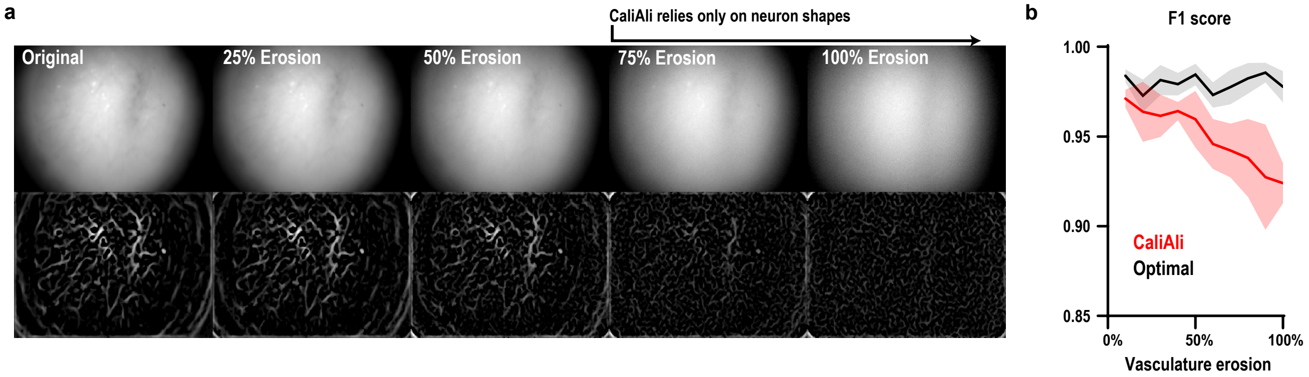

Supplementary Fig. 5 | CaliAli automatically shifts to a neuron-only registration strategy in the absence of BVs. a, To evaluate how CaliAli performs when BVs are faint or missing, we applied increasingly larger gaussian kernels to filter the baseline images in our video simulations. This led to varied extents of BV erosion. **b,** Performance of CaliAli (red) under different levels of vasculature erosion. Optimal plot (black) is the maximum achievable performance by CNMF-E in denoised and perfectly aligned videos. CaliAli effectively utilizes BVs until they are eroded by up to 50%. Beyond this point, CaliAli leans towards a neuron-only registration approach. With total BV erosion, we detected a 5.5% decrease in performance. Shaded regions indicate the 95% confidence interval obtained by BCa bootstrap (n = 8 video simulations).

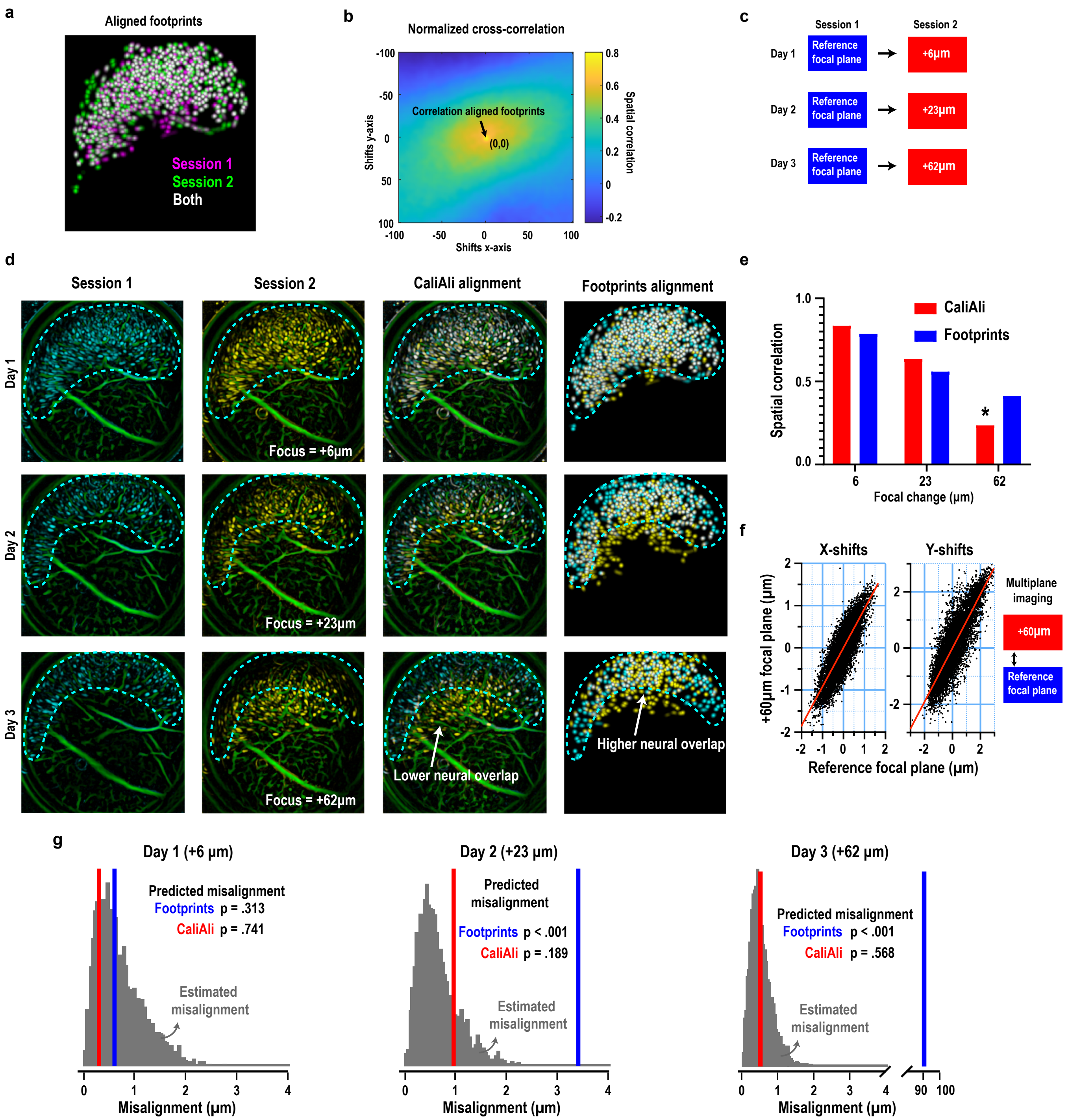

Supplementary Fig. 6 | CaliAli detects large changes in the FOV and produces consistent population activity in the presence of z-axis displacement. a, b, CellReg methodology for identifying mismatched FOVs. **a,** Aligned footprints. **b,** Normalized 2D cross-correlation of the footprints. **c,** Strategy utilized to evaluate CaliAli's performance when sessions in different focal planes are aligned together. **d,** The initial two columns showcase the neuron and BV projections derived from different focal

planes. The subsequent two columns offer a comparative view of alignments achieved by CaliAli versus traditional footprint alignment. Neurons from session 1 are presented in cyan, and those from session 2 are in yellow. Their intersection is visualized in white. BVs from both sessions are combined into the green channel. A dashed cyan boundary indicates the area occupied by session 1 neurons. **e,** Spatial correlation of aligned neuron projections versus footprint projections, with asterisks indicating detected FOV mismatches. **f,** Correlation of xy-shifts between a +60 μm focal plane and a reference focal plane (0 μm) imaged simultaneously with an Inscopix microendoscope. Each point represents the measured x (left) or y (right) shift (in μm) between two frames on the +60 μm plane plotted against the corresponding shift on the 0 μm plane. The red line shows a linear fit (average slope = 0.94), indicating consistent shifts across focal planes. **g,** Inter-session misalignment prediction accuracy for Days 1, 2, and 3 (same as c). Histograms show the distribution of misalignment estimated by motion correction. Red and blue vertical lines indicate the predicted inter-session misalignment values from CaliAli and the footprint-based method, respectively. P-values are the percentile rank of the predicted value within the estimated misalignment distribution. The footprint-based method, but not CaliAli, overestimated inter-session misalignment.

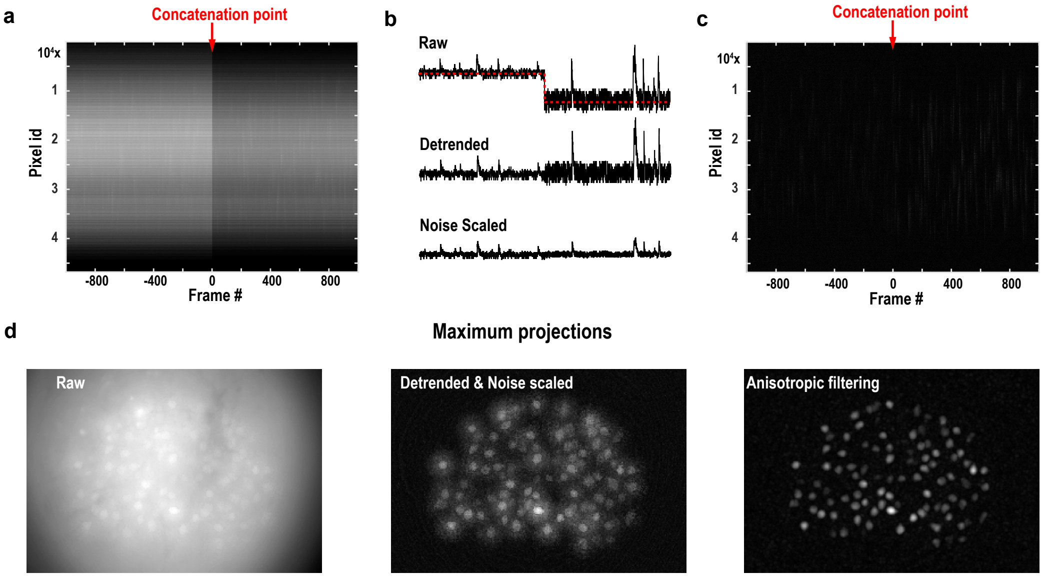

Supplementary Fig. 7 | Detrending and noise scaling minimize concatenation artifacts. a, Display of a two-session concatenated video as a 2D matrix (pixel × time) with artifacts at the concatenation point highlighted by red arrow. **b,** Detrending and noise scaling of each pixel's fluorescence signal. **c,** The improved version of **a** after detrending and noise scaling, illustrating the absence of abrupt pixel intensity changes at the concatenation point. **d,** The video session's maximum projection in its raw state, post-detrending and noise scaling, and after employing the anisotropic filtering and background removal module from MIN1PIPE.

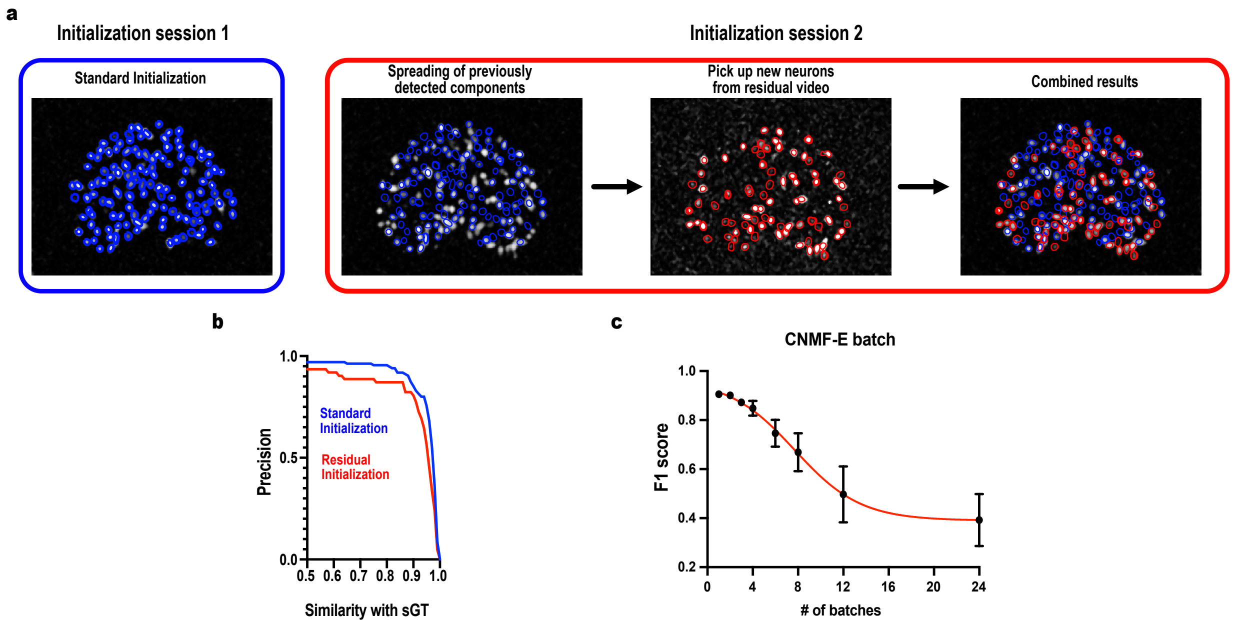

Supplementary Fig. 8 | CNMF-E batch mode propagates errors during initialization. a, Illustration of steps performed during standard neuron initialization by CNMF-E batch. Neurons in the first session are detected using standard CNMF-E implementation. In subsequent batches, neurons are initialized by first propagating the footprints of components detected in previous sessions. Then, new uninitialized neurons are identified in the residual video. **b,** Components extracted from simulated residual videos in subsequent sessions are less precise than those extracted from the raw video by standard initialization (non-batch initialization). **c,** CNMF-E batch performance relative to the number of sessions processed (constant extraction parameters). Although processing a few sessions in batch mode does not substantially reduce performance, the processing of multiple batches propagates errors, leading to a significant decline in extraction performance.

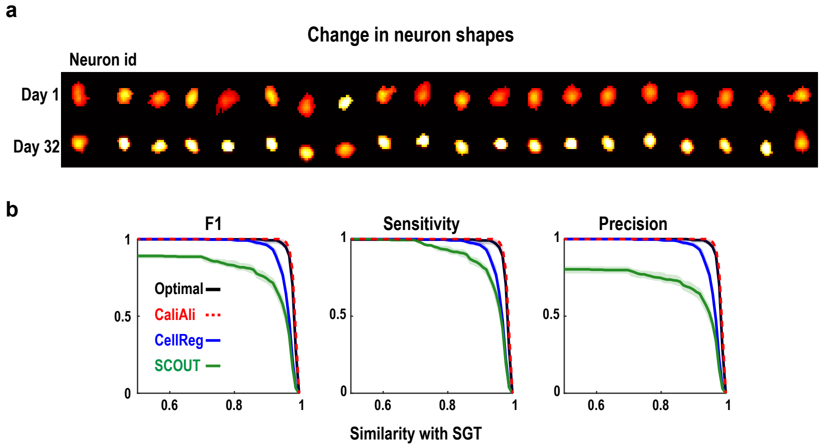

Supplementary Fig. 9 | Assessment of extraction performance considering changes in a neuron's spatial components across sessions. a, To simulate realistic neuron changes across sessions, we simulated Ca^{2+} imaging videos utilizing the spatial components of CA1 neurons imaged 31 days apart. **b,** Incorporating these spatial variations did not compromise performance. For comparison, performance scores from other neuron tracking packages, CellReg and SCOUT, are also displayed. Shaded regions indicate the 95% confidence interval obtained by BCa bootstrap (n = 8 video simulations).

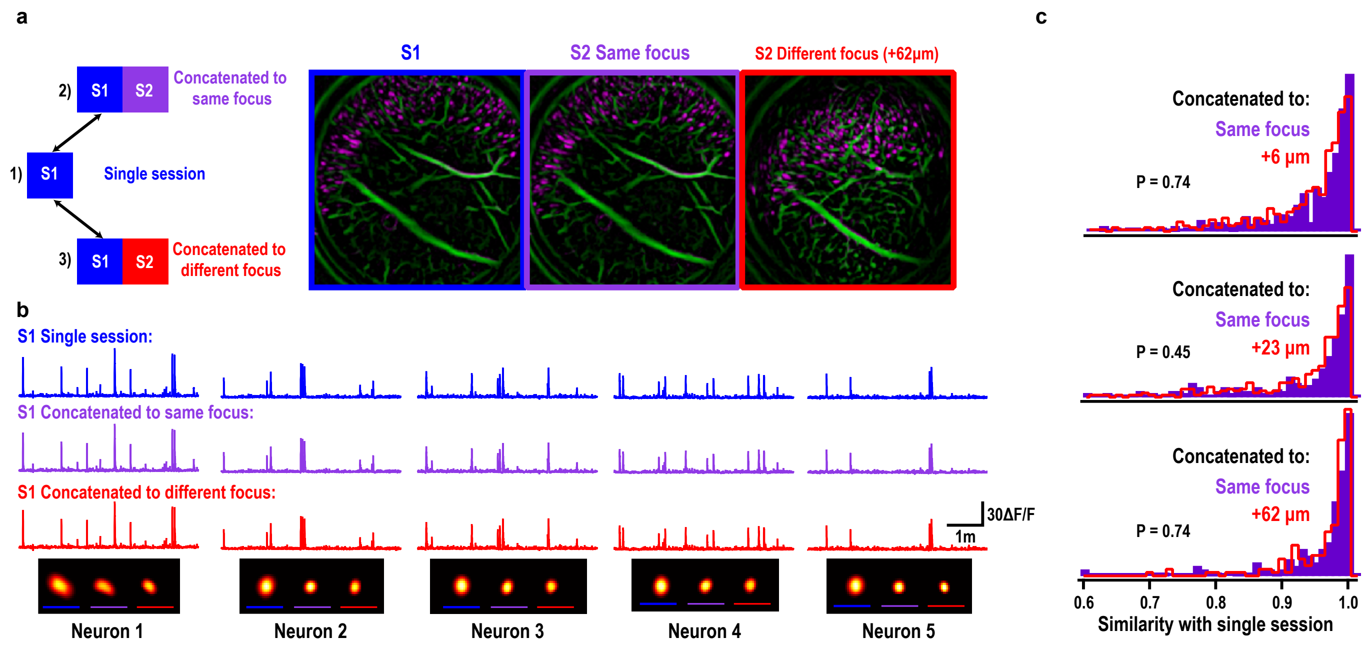

Supplementary Fig. 10 | CaliAli is robust against changes in FOV across sessions. a, To assess whether the extraction of neuronal signals in a session is affected by concatenating that session with another session in a different focal plane, we compared neuron extraction under three scenarios: 1) Independent neuron extraction from one session (S1); 2) neuron extraction from S1 concatenated to another session (S2) in the same focal plane; and 3) neuron extraction from S1 concatenated to S2 in a different focal plane. Focal plane differences of 6, 23, and 62 µm were considered. On the right, BV-neuron projections from S1, from S2 at the same focal plane, and from S2 shifted by +62 µm in the z-axis are shown. **b,** Representative spatial and temporal components corresponding to S1 obtained in the conditions described in a. **c,** Temporal similarities between components extracted independently from S1 (corresponding to the blue traces in b) and from the concatenation of S1 with S2 in the same focal plane (violet traces in b) or a different focal plane (red traces in b). Two-sample Kolmogorov-Smirnov test. Concatenating a session to the same or different focal plane had a similar impact on signal extraction in that session. These results validate that CaliAli is robust against error spread caused by shift in the focal plane across sessions.

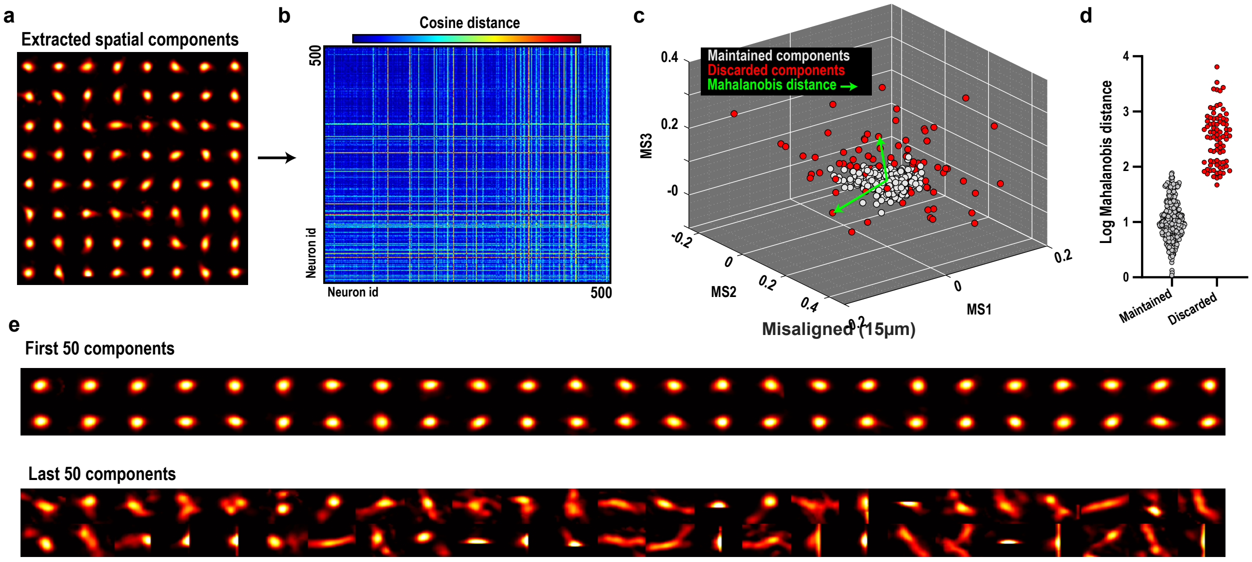

Supplementary Fig. 11 | Streamlined false-positive detection in CaliAli: A semi-supervised and generalizable approach. a, Representative spatial components obtained from a DG recording (64 components out of 500)**. b,** CaliAli automatically sorts components by first calculating the cosine distance matrix between all pairs of spatial components. **c,** Multidimensional scaling of these distances allows the representation of components in a low dimensional space. Components further from the origin are more likely non-somatic. Manually maintained and discarded components are shown in gray and red, respectively. The distance of each component relative to the entire distribution is calculated by the Mahalanobis distance, which is a measure of the distance between a data point and a distribution, taking into account the covariance of the data. It assesses how many SDs a point is from the mean of the distribution. **d**, Scatter plot comparing the Mahalanobis distance between maintained and discarded components. **e,** Spatial components of the first 50 (top) and last 50 (bottom) components out of the 500 extracted after automatic sorting by CaliAli. The latter demonstrates more heterogeneous and irregular patterns indicative of non-neuronal structures.

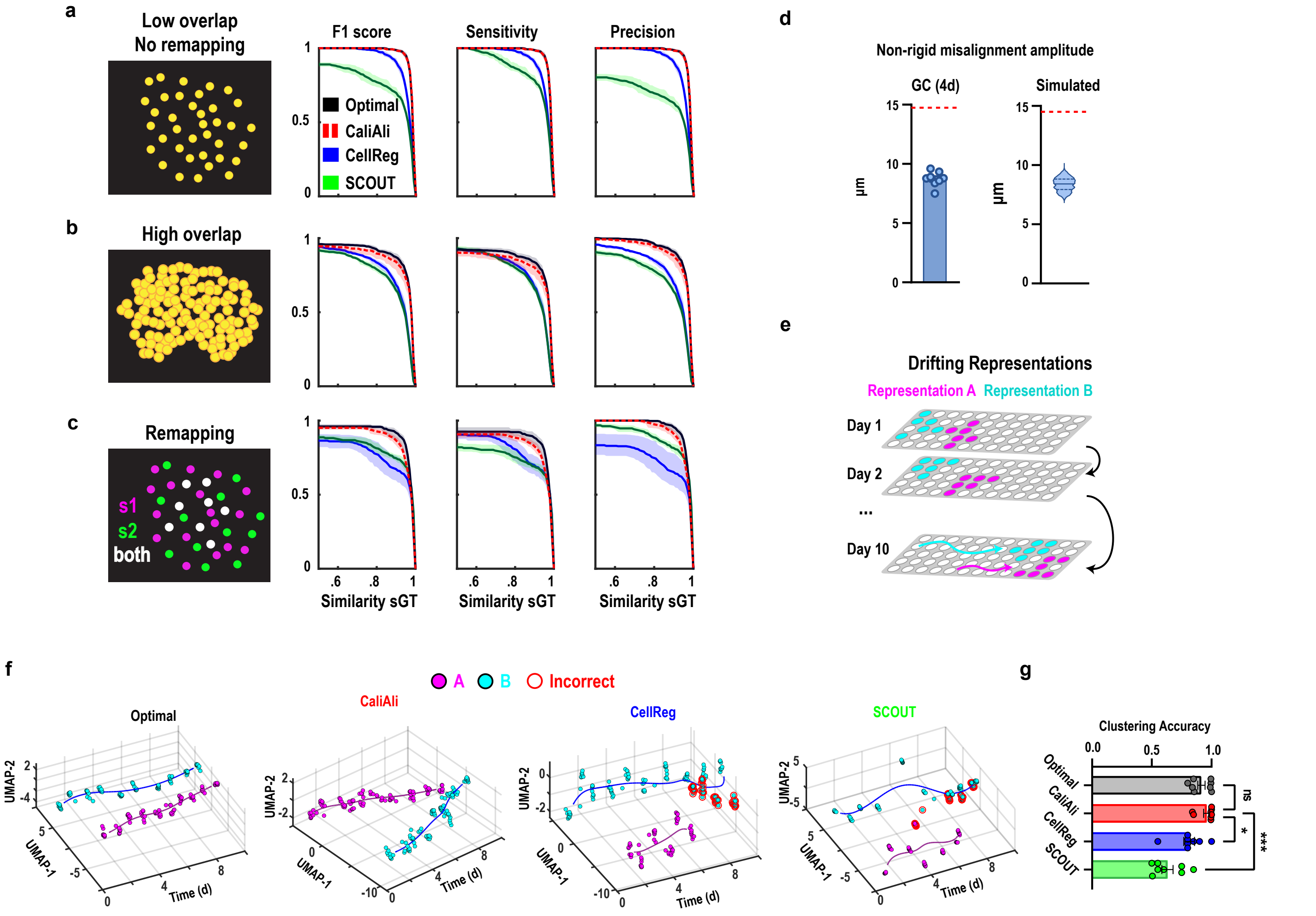

Supplementary Fig. 12 | CaliAli improves neuron trackability in challenging situations common to one-photon Ca^{2+} imaging. a-c, Tracking performance was evaluated in different activity scenarios: a, low overlap, no remapping; b, high overlap; and c, remapping (i.e., change in active neuronal population across sessions). s1 = active in session 1; s2 = active in session 2; both = active in both sessions. Optimal plot (black) is the maximum achievable performance in denoised and perfectly aligned videos. **d,** Empirically estimated and simulated non-rigid misalignment amplitudes. Left data correspond to Supplementary Fig. 1c. **e,** Simulation of a scenario in which active neuron populations gradually drift across sessions but maintain information content. Population activity in response to two different stimuli are shown in magenta and cyan. The neurons recruited by the same stimuli change across days but maintain an orthogonality of representations. **f, g,** Dimensionality reduction and unsupervised clustering with UMAP of Ca^{2+} activity obtained by different methods. Optimal clustering parameters were chosen by clustering simulated ground truth (sGT) data. Repeated measures one-way ANOVA ($F(3, 21) = 17.7$) with Dunnett's multiple comparisons. ***$p < 0.001$, *$p < 0.05$. Error bars = SEM. Shaded regions indicate the 95% confidence interval obtained by bCa bootstrap (n = 8 video simulations).

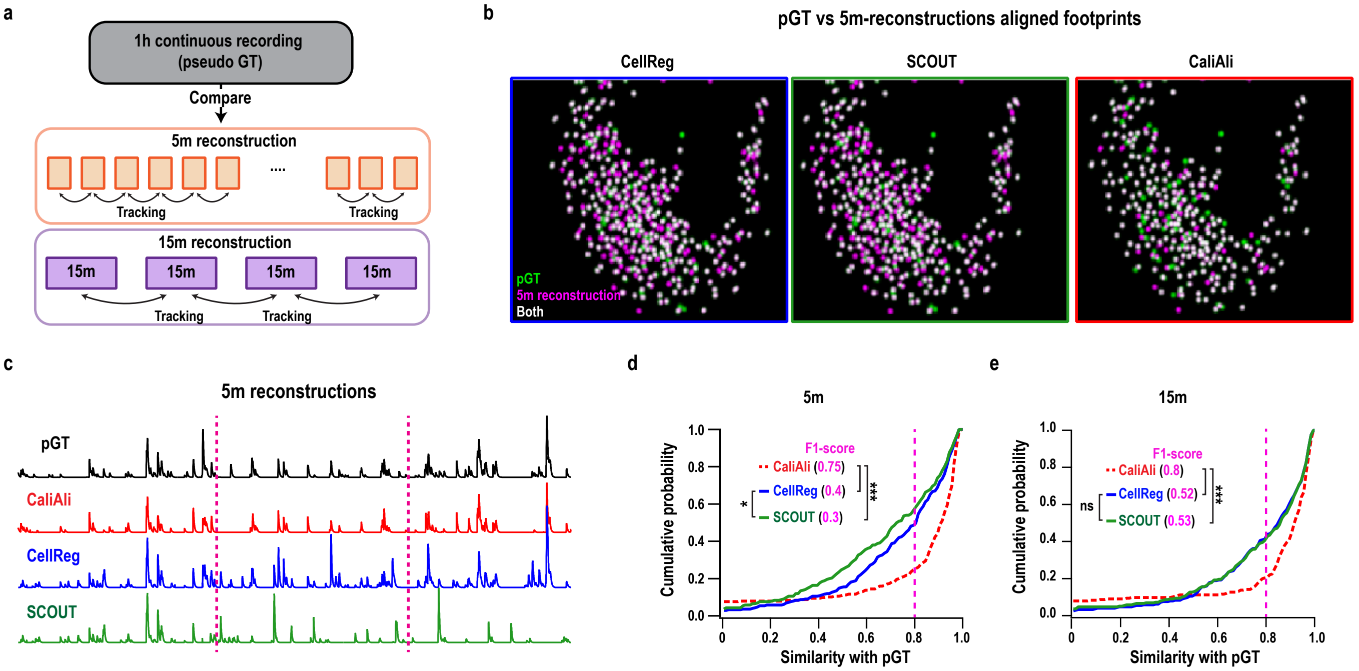

Supplementary Fig. 13 | CaliAli improves neuron trackability across both long and short recording sessions. a, Methodology used to assess the impact of session duration on CaliAli's performance. **b,** Overlay of footprints of the pseudo-ground truth (pGT) and 5-min reconstruction obtained using different tracking methods. **c,** Comparison of a representative pGT Ca^{2+} trace with the 5-min reconstructions obtained using different tracking methods. **d,** Cumulative distribution of the similarity of each neuron obtained from the reconstructed data with the pGT. F1 scores (magenta) were calculated, considering true-positive detections as those with a similarity >0.8 (dashed line). **e,** Same as d, but for 15-min reconstructions. Random permutation test (n = 276 pGT neurons). ***p < 0.001.

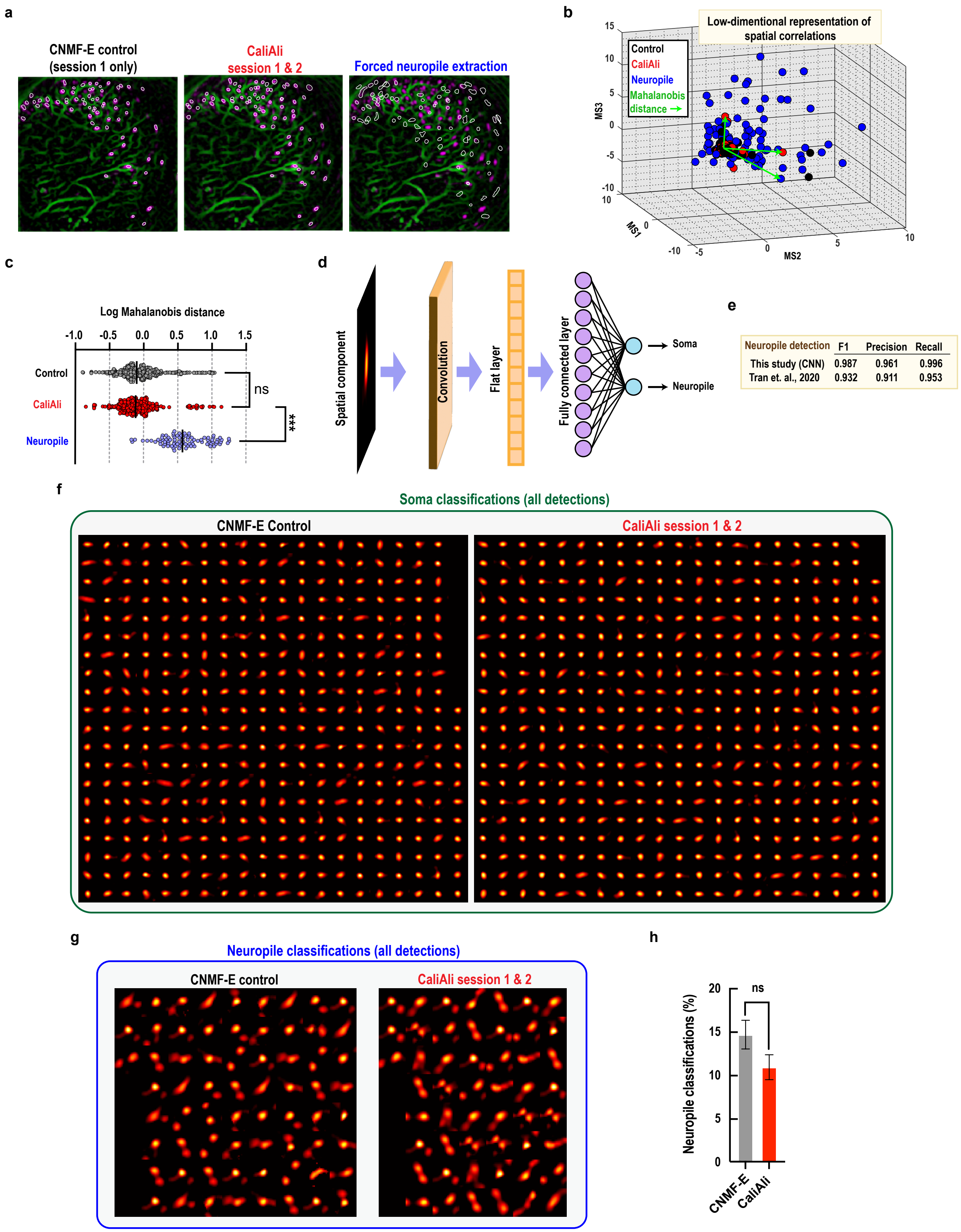

Supplementary Fig. 14 | Increased opto-consistency does not arise from the incorrect alignment of neurons to neuropile structures or the extraction of non-somatic signals. a, BV-neuron projection alongside the spatial outlines of extracted

components (white) obtained using three different approaches: independent extraction of the neuronal signals in each session with CNMF-E (control), extraction from concatenated sessions with CaliAli, and deliberate extraction of neuropile signals. **b**, Low-dimension visual representation of spatial component similarities for each extraction procedure. **c,** Mahalanobis distance of each component relative to the distribution from individually processed sessions. Welch's ANOVA test and Dunnett's T3 multiple comparisons test, $W(2.000, 227.4) = 171.1$, $n = 1478$ components. ***$p < 0.001$. **d,** Convolutional network diagram used to classify somatic and neuropil signals. **e,** F1 and precision classification scores. **f, g**, Spatial components of all ROIs classified as somas or neuropile (all data are shown). **h,** Percentage of neuropile classifications for data obtained with CNMF-E control and CaliAli. Chi-square with Yates correction ($\chi^2(1,927) = 2.181$, $p = 0.140$). Horizontal bars = mean.

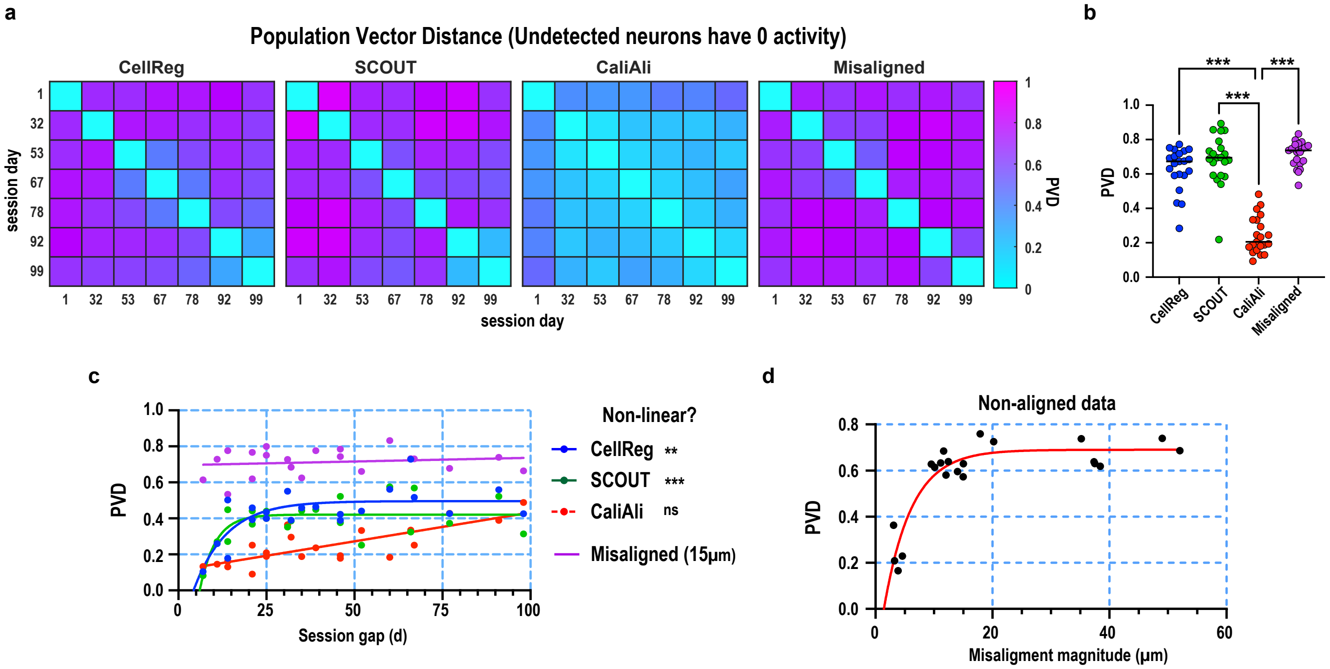

Supplementary Fig. 15 | CaliAli captures the gradual and continuous drifting dynamics of DG population activity. a, b, Same as Fig. 8b, c, but undetected neurons have zero activity. **c,** PVD vs session gap. Data were fitted with an exponential or linear model depending on which was better fitted to the data (F-test). **d,** PVD from data that were not aligned is plotted against the misalignment magnitude estimated with CaliAli. ***p < 0.001, **p < 0.01. Horizontal bars = mean.

Supplementary Note 1: CaliAli can detect dissimilaritiesdissimilarities in the FOV more accurately that other methods.

As depicted in **Fig. 3a**, variations in the FOV can be attributed to shifts in the z-axis. Identifying situations in which there is a substantial change in FOV would be advantageous to correctly interpreting data.

CellReg[1], a prevalent method for footprint alignment, analyzes FOV stability across sessions by comparing the spatial correlation of footprints against those derived from systematic x- and y-axis shifts (2D cross-correlation) (**Supplementary Fig. 6a, b**). If the resultant correlation between aligned footprints does not exceed the median 2D cross-correlation by at least 0.2 units, CellReg issues a cautionary note to indicate dissimilar session characteristics. CaliAli adopts a similar strategy but utilizes correlation images instead of footprints projections. In addition, CaliAli also considers BVs during alignment. Given BV's significant light absorption capacity, pronounced BV structures are discernible even amidst substantial z-axis displacements[2]. Such BV structures can facilitate FOV alignment (in the x/y-axis), especially when active neuronal populations show considerable variation (as would occur with large z-axis displacement). We hypothesize that BVs can prevent the overestimation of neuron overlap. To evaluate neuronal tracking performance in the presence of z-axis shifts, we recorded DG neurons and systematically shifted the focal plane across imaging sessions (**Supplementary Fig. 6c**). We tested small (6 μm), intermediate (23 μm), and large (64 μm) focal shifts. Using this approach, we compared how CaliAli's alignment differs from the alignment of neuronal footprints (**Supplementary Fig. 6d**). Notably, significantly dissimilar FOVs were only detected for large focal displacement and only with CaliAli (**Supplementary Fig. 6e**).

To confirm that footprint alignment methods overestimate neuronal overlap due to unrealistic x-y displacements, we compared their inter-session alignment amplitudes to motion correction amplitudes. For sessions recorded close in time (<5 min), inter-session misalignment should be comparable to motion correction displacements within each session. This holds true even across different focal planes because motion displacements are largely correlated, as demonstrate by multiplane calcium imaging data (**Supplementary Fig. 6f**). We found that CaliAli's calculated misalignment amplitudes matched those observed during motion correction. However, footprint alignment methods significantly overestimated x-y misalignment (**Supplementary Fig. 6g**), indicating that they maximize neuron correlation by introducing unrealistic displacements. CaliAli avoids this by incorporating BV structures, which are more stable across focal planes.

Supplementary Note 2: CaliAli does not compromise extraction performance amidst changes in neuron shape across sessions.

CNMF operates under the assumption that spatial components remain static over time. However, during video concatenation, minor variations in neuron shape might arise. To evaluate how this affects neuronal extraction, we simulated Ca^{2+} imaging videos with progressive changes in neuronal shape across sessions. The extent of these changes was derived from actual data spanning a 32-day period[3] (**Supplementary Fig. 8a**). Notably, CaliAli showed robust neuronal extraction performance against neuron shape changes (**Supplementary Fig. 8b**).

Supplementary Note 3: CaliAli is robust against error spread caused by shift in the focal plane across sessions.

When concatenating multiple sessions, variations in the z-axis across some sessions could compromise extraction in other sessions, as the extraction process is not session-independent. Notably, aligning sessions to a distinct focal plane did not impact CaliAli's neuronal extraction performance in other sessions (**Supplementary Fig. 9a-c**), showing the resilience of the batch method implemented in CaliAli to error spread by z-axis displacement.

Supplementary Note 4: Increased opto-consistency does not arise from the extraction of non-somatic signals.

The extraction of neuronal signals from concatenated videos could result in the inclusion of neuropile signals that would not be included if sessions were processed independently. These signals, influenced by the inherent axial point spread function in one-photon Ca^{+2} imaging, are prone to responding to light stimulation[4]. To rule out this possibility, we compared components extracted by three different procedures: (1) independent extraction of neuronal signals in each session utilizing CNMF-E (control situation in which the concatenation-derived problem would not be present), (2) extraction from concatenated sessions with CaliAli, and (3) deliberate extraction of neuropile signals by initializing neurons in visually neuron-absent regions (**Supplementary Fig. 14a**). If CaliAli does not extract neuropile signals, the shape of extracted components should not be different from those obtained from CNMF-E but should differ from neuropile signals. We represented the spatial similarities between components in a low-dimensional space (see Supplementary Fig. 11 for description of methodology), where components distant from the origin are more likely to constitute non-somatic signals (**Supplementary Fig. 14b**). The morphologies of the components extracted with CaliAli were not significantly different from those obtained by CNMF-E; they were, however, markedly different from those derived from forced neuropile initialization (**Supplementary Fig. 14c**).

As an alternative approach, we also trained a convolutional network to automatically classify somatic and neuropil signals (**Supplementary Fig. 14d**). This model, trained on DG data from eight mice (1,634 annotated components), achieved comparable performance to existing algorithms[5] (**Supplementary Fig. 13e**). Analysis of components extracted by CaliAli and CNMF-E (**Supplementary Fig. 13f, g**; all available components are shown) produced no significant difference in the number of neuropil components detected (**Supplementary Fig. 13h**). Notably, CaliAli identified fewer neuropil components than CNMF-E in both our training data (17.04%) and a previous study in the CA1[5] (21.5%). These insights confirm that CaliAli's high opto-consistency is not a byproduct of neuropile signal extraction.

Supplementary Note 5: | CaliAli improves neuron trackability across both long and short recording sessions.

To further validate that CaliAli's improvement in long-term trackability is not exclusive to short video sessions or sparse brain regions such as the DG, we also tested CaliAli's tracking performance in CA1 recordings. Initially, we conducted continuous Ca^{2+} activity recording for 1 hour, establishing this as a pseudo-ground truth (pGT) dataset. Subsequently, we segmented this recording into sessions lasting 5 or 15 min each. From these segmented sessions, we extracted neuron and tracked them across sessions using CaliAli, CellReg, or SCOUT (**Supplementary Fig. 14a**). We then compared the reconstructed signals from these sessions with the pGT (**Supplementary Fig. 14b, c**). Notably, signals obtained with CaliAli exhibited greater similarity to the pGT dataset compared with other methods, irrespective of whether short or long imaging sessions were utilized (**Supplementary Fig. 14d, e**). Overall, these results suggest that the lower PVD obtained with CaliAli can be attributed to both the alignment and concatenation strategy used and validate that CaliAli can improve neuronal trackability for up to 99 days.

Supplementary Table 1. Differences between CaliAli and other neuron tracking methods.

Features	CalmAn (CNMF-E)	CellReg	SCOUT	CaliAli
Complete pipeline	o	Tracking only	Tracking only	o
Consistent number of neurons tracked across sessions	o	x	x	o
Correction of non-rigid inter-session missaligment	Limited	Limited	Limited	Improved
Correction of rigid inter-session missaligment	o	o	o	o
Optimal neuronal extraction for concatenated recordings	x	Not applicable	Not applicable	o
RAM-efficient neuronal extraction for concatenated recordings	o	Not applicable	Not applicable	o
Align-before-extraction (AE) or extraction-before-aligment (EA)	AE	EA	EA	AE
Incorporation of BVs for aligment	Limited	x	x	o
Multi-session registration strategy (≥3 sessions): motion-correction (MC), reference session (RS), or weighted group-wise (WGW)	MC	RS	RS	WGW
Maximum tested long-term tracking in the original manuscript	n/a	33 days	5 days	99 days
Seamless integration with other calcium imaging pipelines	x	o	x	x
Across sessions tracking validation: simulations (S), place cells (PC), opto-tagging (OT), population vector (PV), manual annotation (MA)	n/a	S, PC	S, PC, MA	S, PC, OT, PV, MA
One-photon (1p)/two-photon (2p) compatible	1p & 2p	1p & 2p	1p & 2p	1p

o = Present , x = Missing

Supplementary Table 2. Animals and viruses

Animals (Jackson Laboratory)	Virus (Addgene)	Source	Target region	Injected coordinates AP, ML, DV (mm):	Experiment	Related data
TIGRE-Ins-TRE-loxP-stop-loxP(LSL)-GCaMP6s	AAV1-Syn-Flex-ChrimsonR-Tdtomato	#62723	DG	-2.0, +1.2, -1.7	Opto-tagging	Fig. 7; Supplementary Fig. 14
C57BL/6J	AAV2retro-CaMKII-0.4-Cre	#105558	DG	-2.0, +1.2, -1.7	Opto-tagging	Fig. 7; Supplementary Fig. 14
C57BL/6J	AAV2retro-cFos-tTA-pA	#66794	DG	-2.0, +1.2, -1.7	Opto-tagging	Fig. 7; Supplementary Fig. 14
C57BL/6J	AAV10-CamKII-GCaMP6f-WPRE	#100834	DG	-2.0, +1.2, -1.7	Parameters for simulated data	Fig. 3; Supplementary Fig. 2
C57BL/6J	AAV10-CamKII-GCaMP6f-WPRE	#100834	DG	-2.0, +1.2, -1.7	Estimation of inter-session missaligment	Supplementary Fig. 1
C57BL/6J	AAV2retro-CamKIIa-jGCaMP8f-WPRE	#176750	DG	-2.0, +1.2, -1.7	Long-term tracking	Fig. 8; Supplementary Fig. 15
C57BL/6J	AAV2retro-CamKIIa-jGCaMP8f-WPRE	#176750	DG	-2.0, +1.2, -1.7	Z-axis changes across sessions	Supplementary Fig. 6, 10
C57BL/6J	AAV2retro-CamKIIa-jGCaMP8f-WPRE	#176750	CA1	-2.0, +1.5, -1.5	Estimation of inter-session missaligment	Supplementary Fig. 1
C57BL/6J	AAV9-hSyn-GCaMP6s-WPRE	#100843	CA1	-2.0, +1, -1.2	1-h continous recording	Supplementary Fig. 13

Supplementary References

1. Sheintuch, L. *et al.* Tracking the Same Neurons across Multiple Days in Ca2+ Imaging Data. *Cell Reports* **21**, 1102–1115 (2017).
2. Song, A., Gauthier, J. L., Pillow, J. W., Tank, D. W. & Charles, A. S. Neural anatomy and optical microscopy (NAOMi) simulation for evaluating calcium imaging methods. *Journal of Neuroscience Methods* **358**, 109173 (2021).
3. Keinath, A. T., Mosser, C.-A. & Brandon, M. P. The representation of context in mouse hippocampus is preserved despite neural drift. *Nat Commun* **13**, 2415 (2022).
4. Zhou, P. *et al.* Efficient and accurate extraction of in vivo calcium signals from microendoscopic video data. *eLife* **7**, e28728 (2018).
5. Tran, L. M. *et al.* Automated Curation of CNMF-E-Extracted ROI Spatial Footprints and Calcium Traces Using Open-Source AutoML Tools. *Front. Neural Circuit.* **14**, 42 (2020).
