# Supplementary material for: A comprehensive suite for extracting neuron signals across multiple sessions in one-photon calcium imaging
Source: Nat Commun. 2025 Apr 11;16:3443. doi: 10.1038/s41467-025-58817-z (PMC11992088; doi:10.1038/s41467-025-58817-z)
Supplement: Supplementary file 2 — Description of Additional Supplementary Files [file 41467_2025_58817_MOESM2_ESM.pdf]

Description of Additional Supplementary Files

Supplementary Video 1: Calculation of Non-Rigid Displacement Using a Multi-Scale Approach with Blood Vessels and Neurons | The upper panels show each projection (blood vessels (green), neurons (red), or both combined (yellow)) in its original form (left) and the corresponding warped version (right) after each alignment iteration. The lower panels illustrate the calculated displacement fields (displayed as displacement grids; left) alongside the Log Demons energy function(right) after each iteration.

Supplementary Video 2: Simulated Ca^{2+} imaging videos used to evaluate CaliAli performance | The video showcases two simulated Ca^{2+} imaging sessions played at 2.5 times speed. Simulated imaging data was created using parameters and baseline images derived from actual calcium imaging data collected from the dentate gyrus.

Supplementary Video 3: Non-rigid misalignments applied to simulated Ca^{2+} imaging data | The video alternates between two average images from different sessions. In one of the sessions, non-rigid misalignments of 10 µm in amplitude were applied.
