# Supplementary material for: A comprehensive suite for extracting neuron signals across multiple sessions in one-photon calcium imaging
Source: Nat Commun. 2025 Apr 11;16:3443. doi: 10.1038/s41467-025-58817-z (PMC11992088; doi:10.1038/s41467-025-58817-z)
Supplement: Supplementary file 6 — Reporting Summary [file 41467_2025_58817_MOESM6_ESM.pdf]

nature portfolio

Corresponding author(s): Masanori Sakaguchi

Last updated by author(s): 2025-01-14

Reporting Summary

Nature Portfolio wishes to improve the reproducibility of the work that we publish. This form provides structure for consistency and transparency in reporting. For further information on Nature Portfolio policies, see our Editorial Policies and the Editorial Policy Checklist.

Please do not complete any field with "not applicable" or n/a. Refer to the help text for what text to use if an item is not relevant to your study.
For final submission: please carefully check your responses for accuracy; you will not be able to make changes later.

Statistics

For all statistical analyses, confirm that the following items are present in the figure legend, table legend, main text, or Methods section.

n/a	Confirmed	
☐	☒	The exact sample size (n) for each experimental group/condition, given as a discrete number and unit of measurement
☐	☒	A statement on whether measurements were taken from distinct samples or whether the same sample was measured repeatedly
☐	☒	The statistical test(s) used AND whether they are one- or two-sided<br>Only common tests should be described solely by name; describe more complex techniques in the Methods section.
☐	☒	A description of all covariates tested
☐	☒	A description of any assumptions or corrections, such as tests of normality and adjustment for multiple comparisons
☐	☒	A full description of the statistical parameters including central tendency (e.g. means) or other basic estimates (e.g. regression coefficient) AND variation (e.g. standard deviation) or associated estimates of uncertainty (e.g. confidence intervals)
☐	☒	For null hypothesis testing, the test statistic (e.g. F, t, r) with confidence intervals, effect sizes, degrees of freedom and P value noted<br>Give P values as exact values whenever suitable.
☒	☐	For Bayesian analysis, information on the choice of priors and Markov chain Monte Carlo settings
☒	☐	For hierarchical and complex designs, identification of the appropriate level for tests and full reporting of outcomes
☒	☐	Estimates of effect sizes (e.g. Cohen's d, Pearson's r), indicating how they were calculated

Our web collection on statistics for biologists contains articles on many of the points above.

Software and code

Policy information about availability of computer code

Data collection	Data was obtained with with Tscope (Srinivasan, et al. BBRC 517.3 (2019): 520-524) using the UCLA miniscope acquisition software (https://github.com/Aharoni-Lab/Miniscope-DAQ-QT-Software)
Data analysis	Data was analyzed with MATLAB R2022a utilizing the code provided in the manuscript.

For manuscripts utilizing custom algorithms or software that are central to the research but not yet described in published literature, software must be made available to editors and reviewers. We strongly encourage code deposition in a community repository (e.g. GitHub). See the Nature Portfolio guidelines for submitting code & software for further information.

Data

Policy information about availability of data

All manuscripts must include a data availability statement. This statement should provide the following information, where applicable:

- Accession codes, unique identifiers, or web links for publicly available datasets
- A description of any restrictions on data availability
- For clinical datasets or third party data, please ensure that the statement adheres to our policy

The data supporting the plots in this study are provided in the Source Data file.
Ca2+ imaging videos, simulation code, and additional source data that exceed the file size limit of the Source Data file are available on Dryad under the accession code: https://doi.org/10.5061/dryad.crjdfn3ck.

Data sets produced in other studies can be obtained according to the data availability statements of the original manuscripts:

Dual-color imaging data: https://doi.org/10.1101/2024.07.03.601770
Place cells data: https://doi.org/10.1016/j.crmeth.2022.100207
The multiplane imaging data used in this study is the property of Inscopix and was used with their permission. This dataset (SampleMultiplane_DS_TPC_data) is available for download to registered Inscopix users through their website: https://iqlearning.inscopix.com/software-downloads/sample-datasets.

The source code for CaliAli, along with demo videos and tutorials, is available on GitHub: https://github.com/CaliAli-PV/CaliAli.
A preserved version of the code at the time of publication has been archived on Zenodo: https://doi.org/10.5281/zenodo.14934924

The code used to create video simulations is available on Dryad under the accession code: https://doi.org/10.5061/dryad.crjdfn3ck.

Research involving human participants, their data, or biological material

Policy information about studies with human participants or human data. See also policy information about sex, gender (identity/presentation), and sexual orientation and race, ethnicity and racism.

Reporting on sex and gender	This study does not involve human participants or human data.
Reporting on race, ethnicity, or other socially relevant groupings	This study does not involve human participants or human data.
Population characteristics	This study does not involve human participants or human data.
Recruitment	This study does not involve human participants or human data.
Ethics oversight	This study does not involve human participants or human data.

Note that full information on the approval of the study protocol must also be provided in the manuscript.

Field-specific reporting

Please select the one below that is the best fit for your research. If you are not sure, read the appropriate sections before making your selection.

[X] Life sciences [ ] Behavioural & social sciences [ ] Ecological, evolutionary & environmental sciences

Life sciences study design

All studies must disclose on these points even when the disclosure is negative.

Sample size	No sample size calculation was performed. Sample selection was based on previous studies benchmarking calcium imaging algorithms (e.g., Jinghao et al., Cell Reports, 2018; Zhou et al., eLife, 2018; Johnston et al., Cell Reports Methods, 2022).
Data exclusions	No data was excluded in this study
Replication	The algorithm's performance was validated using independent datasets from other laboratories and online repositories. These datasets were not part of the development phase of CaliAli.
Randomization	There is no group allocation in this studies. Comparisons were done by analyzing the same data with different algorithms.
Blinding	Due to the nature of this study, which involves a comparative analysis of different algorithms applied to the same dataset, traditional blinding of group allocations was not applicable.

Reporting for specific materials, systems and methods

We require information from authors about some types of materials, experimental systems and methods used in many studies. Here, indicate whether each material, system or method listed is relevant to your study. If you are not sure if a list item applies to your research, read the appropriate section before selecting a response.

Materials & experimental systems

n/a	Involved in the study	
[X]	[ ]	Antibodies
[X]	[ ]	Eukaryotic cell lines
[X]	[ ]	Palaeontology and archaeology
[ ]	[X]	Animals and other organisms
[X]	[ ]	Clinical data
[X]	[ ]	Dual use research of concern
[X]	[ ]	Plants

Methods

n/a	Involved in the study	
[X]	[ ]	ChIP-seq
[X]	[ ]	Flow cytometry
[X]	[ ]	MRI-based neuroimaging

Animals and other research organisms

Policy information about studies involving animals; ARRIVE guidelines recommended for reporting animal research, and Sex and Gender in Research

Laboratory animals	We used TIGRE-Ins-TRE-loxP-stop-loxP (LSL)-GCaMP6s mice along with wild-type mice on a C57BL/6J background. The details of mouse lines used through this study are provided in Supplementary Table 2
Wild animals	No wild animals were used
Reporting on sex	No specific allocation based on sex was performed, as the focus of this study is on algorithm development rather than biological variability
Field-collected samples	This study does not involve field-collected samples.
Ethics oversight	All animal experiments were approved by the University of Tsukuba Institutional Animal Care and Use Committee.

Note that full information on the approval of the study protocol must also be provided in the manuscript.

This checklist template is licensed under a Creative Commons Attribution 4.0 International License, which permits use, sharing, adaptation, distribution and reproduction in any medium or format, as long as you give appropriate credit to the original author(s) and the source, provide a link to the Creative Commons license, and indicate if changes were made. The images or other third party material in this article are included in the article's Creative Commons license, unless indicated otherwise in a credit line to the material. If material is not included in the article's Creative Commons license and your intended use is not permitted by statutory regulation or exceeds the permitted use, you will need to obtain permission directly from the copyright holder. To view a copy of this license, visit http://creativecommons.org/licenses/by/4.0/
